# Helicobacter pylori eradication in the management of glaucoma

**DOI:** 10.22088/cjim.11.2.143

**Published:** 2020

**Authors:** Shahram Ala, Iradj Maleki, Ali Sanjari Araghi, Adeleh Sahebnasagh, Anahita Shahraki

**Affiliations:** 1 Pharmaceutical Research Center, Faculty of Pharmacy, Mazandaran University of Medical Sciences, Sari, Iran; 2 Department of Internal Medicine, Imam Khomeini Hospital, School of Medicine, Mazandaran University of Medical Sciences, Sari, Iran; 3 Department of Ophthalmology, Faculty of Medicine, Mazandaran University of Medical Sciences, Sari, Iran; 4 Clinical Research Center, Department of Internal Medicine, Faculty of Medicine, North Khorasan University of Medical Sciences, Bojnurd, Iran; 5 Pharmaceutical Research Center, Faculty of Pharmacy, Mazandaran University of Medical Sciences, Sari, Iran

**Keywords:** Helicobacter pylori infection, Open-angle glaucoma, Management, Eradication, Intraocular pressure

## Abstract

**Background:**

To investigate the possibility that the eradication of *H pylori* infection is associated with a reduction in the risk of glaucoma.

**Methods:**

Sixty-five successive patients with elevated intraocular pressure (IOP) or glaucoma were included in the study. Serum samples from all subjects were analyzed for the presence of *H pylori*- antibodies using ELISA. Forty patients with positive serologic test were included. Half of the patients enrolled into intervention group and the other half registered as control. Intervention arm was referred to the Gastroenterology Clinic for eradication of *H pylori* and evaluated for the effect of *H pylori* regimen eradication on IOP and glaucoma over 2 months of follow-up. The age-matched controls did not receive treatment. Urea breath test was applied to confirm eradication.

**Results:**

There was a significant (p=0.005) reduction in IOP after complete eradication in the intervention group. This value was not significant in control patients (p=0.08). The mean IOP before treatment of glaucoma in the control group was 23.60±2.37 mmHg and after treatment with anti-glaucoma drugs was 14.25±1.48 mmHg on the onset of study, and 13.55±2.01 mmHg after follow up. The mean IOP before treatment of glaucoma in the intervention group was 24.55±3.6 mmHg and after treatment with anti-glaucoma drugs was 15.15±1.8 mmHg, and 14.3±1.6 mmHg after the eradication of H pylori with a drug regimen. However, after the treatment of glaucoma in all patients, the overall comparison of mean IOP differences showed no statistical difference (P=0.65).

**Conclusion:**

*H pylori* eradication therapy may have a positive effect on the management of glaucoma.

Glaucoma comprises a large group of age-related disorders characterized by progressive optic neuropathy and corresponding visual field defects. It is the second leading cause of blindness globally, after cataracts, and the leading cause of irreversible blindness (1). It is assumed that in glaucoma, the reduction in visual sensitivity occurs as a result of the loss of retinal ganglion cells and their neurons, causing enlargement of the optic disc cup and visual field loss (2). This disease may have a multifactorial cause, which remains largely unknown. Elevated intraocular pressure (IOP) is one of the major risk factors for developing glaucoma, and the goal of medical and surgical treatments for glaucoma is to reduce this pressure. Other risk factors include family history, age, and ethnic background or other medical conditions (3). Since the early manifestation of this disease is asymptomatic or nonspecific in earlier phases, it results in delayed diagnosis with relatively increased medical vigilance throughout the later stages of the disease (4). Helicobacter pylori infection is the most common human infection worldwide (approximately 50% of the world's population is infected) (5, 6). 

Retrospective and prospective studies revealed an association between gastric cancers including lymphoma with *H pylori* infection (70-90%) (7). In various developing countries, more than 80% of the population is *H pylori* positive. The prevalence of *H pylori* in industrialized countries is generally under 40% (8). Some studies have proposed that* H pylori* has been implicated in a variety of diseases that are not related to the gastrointestinal tract, such as chronic urticarial (9), Alzheimer’s (10) and coronary heart disease (11). Besides, several studies have suggested a possible association between this infection and eye diseases, including glaucoma (12).

Several possible theories to explain the pathogenic mechanism underlying this condition have been proposed, since both diseases are more common in older adults (13, 14) and the *H pylori* infection is more common in patients with chronic open-angle glaucoma (15). One of the possible processes in developing glaucoma is vascular disorder and optic nerve ischemia, since chronic ischemia of the optic nerve creates patterns of retinal ganglion cell axonal loss within the optic nerve, similar to glaucomatous optic nerve damage. Chronic *H pylori* infection may induce a strong systemic host immune response and release of various vasoactive and proinflammatory substances as well as influencing the apoptotic process. Therefore, it may cause systemic oxidative stress and damage to the trabecular meshwork and optical nerve head which results in elevated IOP and glaucoma (12). Besides, several studies have investigated the potential role of *H pylori *in the pathogenesis of atherosclerosis and increase in platelet aggregation (16, 17).

Whether exposure to this bacterial agent promotes the initiation and progression of glaucoma is still controversial. Therefore, we investigated a possible association between *H pylori* infection and glaucoma by evaluating clinical parameters in glaucoma (18). We also explored the possibility that eradication of *H pylori* may be associated with a reduced risk of glaucoma. 

## Methods

***Participants:*** Sixty-five successive patients who referred to an ophthalmology clinic were included in the study. All participants underwent a complete clinical examination for diagnosis of glaucoma, including measurement of intraocular pressure with applanation tonometry, visual field examinations with Humphrey perimeter, examination of the optic nerve using a slit-lamp biomicroscope with a 90 diopter lenses, and vision testing with Snellen chart. Additionlly, the differential diagnosis of open-angle and angle-closure glaucoma was performed by applying 3 mirror gonioscopy lens on all the patients by an ophthalmologist. 

All the patients who were evaluated in the study had an elevated intraocular pressure or glaucoma. They were also found to have negative history of all kinds of eye diseases except glaucoma and diabetes mellitus, and did not take drugs influencing intraocular pressure (e.g. anticholinergic, carbonic anhydrase inhibitors, long-term use of glucocorticoids).

All patients received verbal and printed information, and all provided written consent before entry into this study. The study protocol was approved by the Ethics Committee of Mazandaran University of Medical Sciences. 

***Study Design:*** In the present study, patients were evaluated for the effect of administration of *H pylori* eradication on intraocular pressure and glaucoma over a 2 months follow-up period. After a detailed history and complete examination taken from all patients with glaucoma and an assessment of their IOPs, they were referred to a diagnostic laboratory for the primary serologic tests of *H pylori*. Venous blood samples were drawn from each patient for serologic testing for *H* *pylori* IgG antibodies. Serum samples were stored at -20^օ^C for analysis. Forty patients with positive serologic test were included. Half of the patients enrolled to intervention group and the other half registered as control. Henceforth, the intervention group referred to Gastroenterology Clinic for treatment and eradication of *H pylori*. The 20 age-matched controls did not receive treatment. Only topical glaucoma treatment was used in this study and topical medications for glaucoma treatment were similar in both groups during study follow-up. The ophthalmologist was masked to the *H pylori* status of the patients. 

***H Pylori Serologic Testing:*** Helicobacter pylori serologic testing was evaluated using a commercial enzyme-linked immunosorbent assay technique (Trinity serologic kits, manufactured by Biotec Company of USA). The manufacturer’s recommended cut off value was applied to determine patient's serologic finding as positive or negative.

***Treatment of H Pylori Infection: ***The eradication regimen of *H pylori* infection included a two-week course of omeprazole (20mg twice a day), amoxicillin (1 g twice a day), metronidazole tablets (500 mg twice a day), and bismuth (240 mg four times a day). The patients were given verbal and written instructions to take into account the importance of taking medications regularly, and to record possible adverse effects during the treatment course and their compliance to the therapy. They were also advised not to stop their therapeutic regimen and call the physician if they experienced severe side effects. Compliance was evaluated by counting medication after therapy. Four weeks after the end of the treatment course and in order to confirm eradication, the subjects underwent a urea breath test (UBT) by a gastroenterologist. 

The patients were advised not to consume antibiotics, orally or parenterally, or drugs that reduce gastric acid secretion (PPIs, H_2_ blockers…) during these four weeks before undergoing the UBT. Those with therapeutic-failure, as a result of microbial resistance or noncompliance, underwent another 14 days of *H pylori* treatment with the alternative four-drug regimen as follows: omeprazole (20 mg twice a day), amoxicillin (1g twice a day), clarithromycin (500 mg twice a day), and bismuth (240 mg four times a day). Once more, 4 weeks later, an assessment of *H pylori* eradication with UBT was done for this group of patients. At the time, *H pylori *eradication was proven, the subjects were referred to the Ophthalmology Clinic. The study participants had their IOP measured. All changes were recorded and compared with former values.

**Statistical Analysis:** Qualitative variables were reported by frequency and percentage, and quantitative variables were reported by mean±SD. The data was assessed by Pearson chi-square test for qualitative parameters, and t-test for quantitative parameters. All statistical analysis was conducted using SSPS software Version 17 (SPSS Inc., Chicago, IL, USA), and differences with a value of p<0.05 were considered significant. The statistician analyzing the final results was blind to the assignment of the patients.

## Results

***Participants: ***Demographic and baseline clinical characteristics of patients are presented in table 1. Among the 65 glaucoma patients who were eligible candidates for participating in the study, 25 patients were excluded, 20 patients due to their negative serologic test, 4 patients declined to participate and one passed away. In total, 69.2% of the patients tested positive for serologic test, and 40 patients with elevated IOP and a positive serologic test for *H pylori* were included in the study. Patient characteristics in two study arms were similar and there was no significant difference between the two groups in demographic and clinical characteristics (age, gender ratio, concomitant comorbidities). 

The mean age of the patients was 61.3±11.2 years in intervention group and 62.95±8.31 years in control group. The eradication rate was 55% for the first course of regimen, and the treatment of *H pylori* was positive (100%) in all the 40 patients after they had received the second phase of eradication therapy. All patients were compliant and responsive with the study by the number of medications remaining after eradication therapy. Adverse effects were mild and tolerable and included abdominal pain, nausea or vomiting, and diarrhea. 

**Figure 1:**
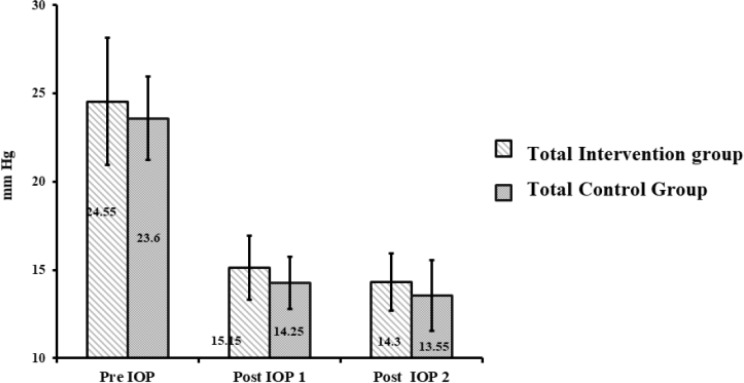
The comparison of IOP differences of all of the patients in intervention and control groups

Nevertheless, all the patients completed the trial and no serious adverse experience was reported. Table 2, figure 1 illustrate IOP in all glaucomatous patients (n=40) at baseline, after treatment with anti-glaucoma drugs and after 2 months of follow-up.

**Table 1:** Baseline and laboratory characteristics of patients

**Characteristics**	**Intervention (n=20)**	**Control (n=20)**	***P*** **-value**
Age, mean±SD (absolute range), y	61.3±11.2	62.95±8.31	0.78
Male sex, No. (%)	9 (45%)	8 (40%)	0.8
**Concomitant Condition, No(%)**			
Concomitant disease	16 (80)	13 (65)	
Hypertension	4 (20)	3 (15)	
Diabetes	3 (15)	5 (25)	
Hypertention & diabetes	5 (25)	1 (5)	

**Table 2:** Comparison of mean intraocular pressure parameter for glaucoma cases at baseline, after treatment with anti-glaucoma drugs and after 2 months of follow-up

	**Mean±SD Measurement value**			
	**Baseline**	**Treatment with anti-glaucoma drugs**	**2-month**	***P*** **-value**
**Patient Group** **Intraocular Pressure, mmHg** Intervention Control	24.55 ±3.6 23.60 ±2.37	15.15 ±1.8 14.25 ±1.48	14.3 ±1.6 13.55 ±2.01	0.65
**Male** **Intraocular Pressure, mmHg** Intervention Control	25 ±3.11 24.30 ±2.31	15 ±2.39 14.25 ±1.48	13.88 ±1.64 13.60 ±2.11	0.35
**Female** **Intraocular Pressure, mmHg** Intervention Control	24.25 ±4.02 22.90 ±2.33	15.25 ±1.42 14.30 ±1.49	14.58 ±1.62 13.50 ±2.01	0.76

***The comparison of IOP differences of all patients in both intervention and control groups: ***The mean IOP before treatment of glaucoma in the control group was 23.60±2.37 mmHg and after treatment with anti-glaucoma drugs was 14.25±1.48 mmHg on the onset of the study and 13.55±2.01 mmHg after 2 months follow up. Their difference was 0.7±0.92 mmHg. The mean IOP before treatment of glaucoma in all the patients receiving *H pylori* treatment was 24.55±3.6 mmHg and after treatment with anti-glaucoma drugs was 15.15±1.8 mmHg. It was 14.3±1.6 mmHg after *H pylori* eradication with drug regimen, with the difference being 0.85±1.18 mmHg. In the intervention group, IOP decreased significantly after eradication (P=0.005); but this value was not significant in the control group (p=0.08). However, after the treatment of glaucoma in all patients, the overall comparison of mean IOP differences between the control and intervention groups showed no statistical difference (p=0.65). 

## Discussion

Glaucoma is the second leading cause of irreversible blindness in the world. It is usually accompanied with a raise in intraocular pressure. Unfortunately, because of lack of accurate information on the prevalence of glaucoma and its resulting blindness in our country, as well as, the irreversibility of its adverse effects, it is necessary to focus more on prophylactic procedures (19). The only proven preventive strategy to attenuate optic damage is to lower the IOP through ocular hypotensive therapy (20). Pharmacologic treatment for glaucoma involve reducing the formation of aqueous humor or allowing fluid to flow out of the eye. However, the efficacy of these medical interventions is only 20-30% (21). 

Therefore, a rational approach to the management of glaucoma involves focusing more on modifying risk factors associated with elevated IOP, which may help prevent glaucoma progression. In previous studies, a higher prevalence of *H pylori* in OAG was documented. They concluded that *H pylori* infection is much more prevalent in patients with primary open angle glaucoma (22-24). This study identified additional information of a link between *H pylori* and glaucoma. The result of the current study showed that IOP decreased significantly after eradication therapy in the intervention group. Whereas, this was not the case in the control group, regardless of the similar topical therapy received throughout the study. However, the overall mean IOP did not differ statistically between the groups after administration of anti-glaucoma eye drops. 

In the present study, we obtained a complete eradication rate of 100% by using two courses of quadruple eradication regimen of 14 days. Fewer eradication rates have been achieved in other studies (23). 

Strong evidence supports the positive impact of controlling IOP and slowing down the process of optic disc damage and narrowing of the visual field (25). The mean IOP before treatment with anti-glaucoma medications was 24.3±5.6. The normal range of IOP is 10-24 mmHg. In primary open-angle glaucoma, IOP generally does not exceed 30 mmHg. In our study, the mean IOP after the administration of anti-glaucoma medicine was 15.15±1.8 mmHg. In a similar study by Kountoras et al, the mean IOP after treatment was 18.91±3.6 (23). 

In patients with severe optic disc damage or focal narrowing of retinal vessel, it is recommended to lower IOP by a substantial amount, preferably less than 15 mmHg. In our study, after eradication of *H pylori*, the mean IOP decreased to 14.3±1.6. This is about 0.85 mmHg decrease in IOP in a 2 month’ follow-up. This amount was 17.84±2.3 in Kountoras' study and lowered as much as 1.07 mmHg after two years of follow-up (23). Although this significant amount of reduction may not seem clinically important, we should consider the slow, potentially progressive nature of glaucoma. Therefore, a longer term follow-up is required to further evaluate the efficacy of this modality. From a total of 40 patients in our study, women were disproportionately affected by glaucoma, representing 55% of all people; the greater number of women affected may be derived from their greater longevity (19). 72% of the study participants had medical comorbidity including diabetes and hypertension, frequent coexisting risk factors for the development of POAG (26, 27). 

Some studies suggest a higher prevalence of glaucoma in patients with vascular disorders such as arterial hypertension and diabetes. It should be taken into account that anti-glaucoma medicine (e.g, topical beta-blockers) may adversely affect the background comorbid medical illnesses in this population (e.g, by increasing serum lipid level) (28, 29). In this study, the mean age of the patients was 61.3±11.2 years in the intervention group and 62.95±8.31 years in the control group. In a study by Abrishami et al. the mean age of the study population was 60.8±20.6 years in the intervention group and 66.0±19.8 years in the control group (30). They reported the prevalence of seropositivity as 70.5%, which is similar to the current study’s (69.2%). In another study carried out by Galloway, the mean age among the study population was 65.8 years; however, seropositivity was much lower, measuring 26.3% in the POAG group (22). 

An enzyme-linked immunosorbent assay (ELISA) for the quantitative detection in serum of the IgG antibodies to *H pylori* was performed like Galloway and Abrishami study (22, 30). This serodiagnostic assay provides excellent sensitivity and specificity (96% and 93%, respectively) (31). Serologic test is widely available, non-invasive and inexpensive, and quick to perform. In our study, we applied the urea breath test to document post-treatment eradication. This method is an accurate and less invasive test with good sensitivity and specificity (93% and 98%, respectively) (31). In a similar research study by Kountouras, the patients underwent upper GI endoscopic procedure for histologic testing. Although this is considered the gold standard for the diagnosis of active *H pylori* infection, this test is invasive and the procedure itself could result in an emergency (23, 32). Treatment failure can certainly be a result of antibiotic-resistant and non-compliance. Failure in the first course of treatment indicate antibiotic-resistant pathogens. Therefore, metronidazole was replaced with clarithromycin in the eradication regimen and therapy continuing another 14 day course. Despite the small sample size, eradication therapy may improve the outflow facility of the eye. Although the results of this pilot study give researchers hope of finding new modalities for this population, further studies with larger group of patients and longer follow-up period are needed to generalize our results.

It should be noted that *H pylori *eradication regimen are not listed as medication changing IOP in literature. However, a third control group of *H pylori* positive patients receiving eradication regimen and monitored for changes in IOP could exclude this confounding factor and it would be suggested that future studies consider this second control group.

In conclusion, the result of the current study showed that *H pylori* eradication therapy may have positive effects on better management of primary open-angle glaucoma and suggest a possible link between *H pylori* and glaucoma.
